# Elevated B-type natriuretic peptide levels in pregnant women with hyperemesis gravidarum: a biomarker of cardiac stress?

**DOI:** 10.1590/1806-9282.20242031

**Published:** 2025-08-08

**Authors:** Mustafa Bakırcı, Nagihan Sarı, Ethem Serdar Yalvaç, Ayşe Yeşim Göçmen

**Affiliations:** 1 Yozgat Bozok University, Faculty of Medicine, Department of Obstetrics and Gynecology – Yozgat, Turkey.; 2 Hüma Hospital, Department of Obstetrics and Gynecology – Kayseri, Turkey.; 3 Yozgat Bozok University, Faculty of Medicine, Department of Biochemistry – Yozgat, Turkey.

**Keywords:** BNP, Cardiac output, Hyperemesis gravidarum, Pregnancy

## Abstract

**OBJECTIVE:**

The aim of this study was to evaluate serum B-type natriuretic peptide levels in pregnant women with hyperemesis gravidarum and compare them with healthy pregnant women.

**METHODS:**

In this prospective cross-sectional study, sample size calculation using G*Power determined a minimum of 40 participants per group, totaling 80 participants. The study included 43 pregnant women with hyperemesis gravidarum and 43 healthy pregnant controls. Hyperemesis gravidarum diagnosis was based on clinical and laboratory criteria, including weight loss, ketonuria, and electrolyte imbalance. Exclusion criteria included pre-existing cardiovascular or systemic diseases, multiple pregnancies, and smoking. Serum B-type natriuretic peptide levels and various biochemical parameters were measured using standard laboratory techniques. Statistical analyses were performed using the Statistical Package for the Social Sciences software, with a p-value of <0.05 considered statistically significant.

**RESULTS:**

B-type natriuretic peptide levels were significantly higher in the hyperemesis gravidarum group compared to the control group (HG: 9.6±2.5 pg/mL [95%CI 8.9–10.3]; control: 8.1±1.8 pg/mL [95%CI 7.5–8.6]; Cohen's d=0.70, p=0.016). No significant differences were found between the groups in terms of age, body mass index, and other biochemical parameters, including hemoglobin, electrolytes, and inflammatory markers. B-type natriuretic peptide levels were not significantly correlated with gestational week, maternal age, body mass index, or hemoglobin levels.

**CONCLUSION:**

In this study, we found that B-type natriuretic peptide levels are elevated in hyperemesis gravidarum, and we believe that this may be associated with increased cardiac stress. B-type natriuretic peptide may serve as a potential biomarker for monitoring cardiovascular changes in hyperemesis gravidarum. Further research is warranted to validate these findings and explore the role of B-type natriuretic peptide in the clinical management of hyperemesis gravidarum.

## INTRODUCTION

Up to 85% of pregnant women experience nausea and vomiting (NPV) during pregnancy, but severe hyperemesis gravidarum (HG) affects 0.3–3% of pregnant women^1^. Women with HG may experience dehydration and weight loss and may require hospitalization. The exact cause of HG is not fully understood and is thought to be multifactorial. Factors such as pregnancy at a young age, female fetus, multiple pregnancies, molar pregnancy, and other underlying conditions (thyroid disorders, type 1 diabetes, hypercholesterolemia, etc.) increase the risk of HG^2,3^. Currently, there is no valid biomarker for diagnosing or ruling out HG^4^. Therefore, it is a clinical diagnosis that can be made after other causes of NPV have been excluded. In the latest guidelines of the Royal College of Obstetricians and Gynaecologists, HG is defined by >5% weight loss, dehydration, and electrolyte imbalance compared to pre-pregnancy levels^5^. According to the recently published Windsor definition for HG diagnosis, symptoms must begin before the 16th week of pregnancy, with severe nausea or vomiting, an inability to eat or drink normally, and limitations in daily activities^6^.

Nausea and vomiting are common symptoms in early pregnancy and are typically considered signs of a healthy pregnancy^7^. If untreated, HG can lead to Wernicke's encephalopathy, central pontine myelinolysis, liver dysfunction, and renal failure. HG may also result in preterm birth and low birth weight^8^. Hormonal, immunological, and psychological theories have been proposed regarding the etiology, but uncertainties remain^1^. Recent studies have identified that growth differentiation factor 15 (GDF-15) is produced in trophoblast cells early in pregnancy and binds to receptors in the brain's "vomiting center," the area postrema^9,10^. This has been proposed as one of the key links between early placental growth and nausea and vomiting in pregnancy^9^. Additionally, it has been found that individuals with a family history of HG are at increased risk of developing this condition. Recent research suggests that this risk may be related to single-nucleotide polymorphisms in the GDF15 gene and the gene encoding the receptor in the area postrema^11^. Additionally, GDF-15 has been reported to be involved in inflammatory pathways, associated with cardiac stress, and a potential biomarker of chronic cardiovascular overload^12^. Its level has been found to change in response to hemodynamic alterations^13^. In this regard, it is involved in similar pathways to BNP.

BNP is released by cardiac myocytes in response to left ventricular load^14^. Elevated BNP levels can be used as a biomarker to indicate the risk of cardiovascular complications. BNP levels rise in conditions such as heart failure, acute coronary syndrome, pulmonary hypertension, chronic hypertension, chronic kidney failure, and heart valve diseases, and may also be influenced by factors such as physical activity, age, and gender^15^.

BNP reduces vascular tone and induces natriuresis and diuresis. Physiological changes in pregnancy can alter the concentration of natriuretic peptides. Pregnancy is associated with a 50% increase in cardiac output, ventricular remodeling, and significant, progressive plasma expansion. Additionally, there is at least a 50% increase in glomerular filtration rate. These hemodynamic changes may cause fluctuations in natriuretic peptide levels^16^. Studies have shown that BNP levels remain stable in normal pregnancies. However, in preeclampsia, a heart disease that arises due to stress on the ventricles of the heart during pregnancy, BNP levels increase^17^.

The characteristic physiological hemodynamic changes in pregnancy include increases in blood volume, cardiac output, and heart rate. These changes can unmask underlying heart disease or worsen existing cardiac conditions, potentially leading to heart failure^18^.

BNP is considered one of the most appropriate natriuretic biomarkers reflecting the cardiovascular system, as it is associated with heart failure, increased ventricular stress, and congestive disorders^19^. Due to physiological changes related to pregnancy, troponin levels and diagnostic tools such as echocardiography may vary even in uncomplicated pregnancies. This variability may be insufficient for assessing maternal cardiac sufficiency and adaptation during pregnancy^20^. For these reasons, we considered BNP a suitable alternative for evaluating hemodynamic changes in HG.

We hypothesize that BNP levels are elevated in HG due to volume loss and could serve as a potential biomarker for HG-related cardiac stress.

## METHODS

### Study design

This is a cross-sectional, prospective study. Ethical approval was obtained from the Institutional Review Board of Bozok University Faculty of Medicine (Non-invasive Clinical Research Ethics Committee, protocol number 12.04.2016/39), and informed consent was obtained from all participants. The study included pregnant women aged 18–35 who presented to the obstetrics and gynecology outpatient clinic with HG during the first trimester. This age range was chosen to prevent maternal age from being an independent variable (as cardiovascular changes, hypertension, and gestational diabetes are more common in older mothers) and to ensure a homogeneous study group. Additionally, only those who had not previously required intravenous medication or hospitalization were included. A control group was formed from pregnant women without nausea and vomiting, matched by gestational week, maternal age, and BMI (body mass index) to the HG patients. HG diagnosis was determined based on the presence of at least two of the following criteria: (i) weight loss of at least 2.25 kg, (ii) ketonuria >80 mg/dL in a random urine sample, (iii) hypokalemia (potassium <3.0 mEq/dL) or hyponatremia (sodium <134 mEq/dL) requiring intravenous replacement, and (iv) more than two visits to the Obstetric Emergency Service due to symptoms associated with HG^21^. Exclusion criteria included a history of ischemic heart disease, valvular heart disease, hypertension, chronic kidney failure, diabetes mellitus, thyroid disorders, gastrointestinal diseases, liver diseases (affecting liver enzyme levels), molar pregnancy, multiple pregnancies, recurrent pregnancy history, and smoking. Additionally, patients with lung diseases, infectious diseases, and psychiatric disorders such as anxiety disorders, panic attacks, and depression, which could potentially cause elevated BNP levels, were excluded. The ethnic background, age, height, weight, gravida, parity, and gestational week of all included pregnancies were recorded. The gestational age was determined based on the last menstrual period and first-trimester ultrasound examination. The serum levels of BNP, hemoglobin, hematocrit, white blood cells, platelets, fasting blood glucose, urea, creatinine, lipid profile, aspartate aminotransferase (AST), alanine aminotransferase (ALT), sodium, potassium, chloride, C-reactive protein (CRP), and thyroid-stimulating hormone (TSH) were measured. Blood samples were collected in vacationer tubes containing ethylenediaminetetraacetic acid (BD, UK).

### Analytical methods

In this study, participant identities were blinded, and each participant was assigned a unique identification number to maintain confidentiality and minimize bias. All blood samples were centrifuged at 1509*g* for 10 min, and the sera were stored at −80°C until analysis by a blinded technician using a double-blind method, in accordance with the clinical data of the patients. For BNP measurement, commercial enzyme-linked immunosorbent assay kits were used (ELABSCIENCE, China). The measurements were carried out using a microplate reader (Bio Tech Instruments, EL×800^TM^, USA) at standard wavelengths, following the test instructions and using a blinded method. All other parameters were measured using standard laboratory techniques in the core laboratory.

### Sample size calculation

In this study, the required sample size was calculated to evaluate the difference in means between two independent groups. An a priori power analysis was conducted using the G*Power 3.1 software, based on a one-tailed t-test. The parameters for the analysis included an effect size of d=0.75 (medium to large effect), a significance level (α=0.05), a statistical power (1-β=0.95), and an equal allocation ratio (N2/N1=1). The analysis determined that a minimum of 40 participants per group, for a total of 80 participants, was required. The calculated actual power for this sample size was 1-β=0.954.

### Statistical analysis

Statistical analysis was performed using the Statistical Package for the Social Sciences (SPSS) 27.0 software package (IBM Corporation). Continuous variables were presented as mean±standard deviation or median, and categorical variables were presented as percentages. A p-value of <0.05 was considered statistically significant. The χ^2^ test was used to compare proportions. The normality of continuous variables was analyzed using the Kolmogorov-Smirnov test. For normally distributed continuous variables, comparisons between independent groups were made using the Student's t-test; otherwise, groups were compared using the Mann-Whitney U test. Correlation analysis was performed to determine the correlation between (a) BNP and BMI, (b) BNP and maternal age, (c) BNP and gestational week, and (d) BNP and hemoglobin. Variables with a p-value <0.05 in the group comparisons were entered into the multivariate analysis model and selected using a stepwise selection procedure.

## RESULTS

A total of 43 pregnant women with HG and 43 pregnant women without HG from the same ethnic background were included in the study. There were no statistically significant differences between the groups in terms of age, BMI, and pregnancy history (Table 1).

**Table 1:** Analysis of demographic and clinical data of the patient and control groups.

Variable		Control (n=43)	HG (n=43)	p-value
Age (years)		27.84±5.21	26.1±5.1	0.739
Body mass index		26.69±5.12	25.53±4.41	0.257
Gestational age (weeks)		8.7±3.06	9.3±2.59	0.073
Gravida		2.49 (1–6)	2.35 (1–7)	0.568
Parity		1.16 (0–4)	0.95 (0–3)	0.135
Number of abortions		0.21 (0–2)	0.28 (0–2)	0.283
Dilation and curettage (D&C)		0.07 (0–1)	0.05 (0–1)	0.362
Number of living children		1.16 (0–4)	0.95 (0–3)	0.123
Education level				0.267
	Illiterate	1 (2.3%)	0 (0 %)	
	Primary school	15 (34.9%)	23 (53.5%)	
	Middle school	21 (48.8%)	12 (27.9%)	
	Elementary education	36 (83.7%)	25 (81.4%)	
	High school	5 (11.6%)	6 (14.0%)	
	University	0 (0%)	1 (2.3%)	
	Graduate degree	1 (2.3%)	1 (2.3%)	
Socioeconomic status				0.004
	Low	23 (53.5%)	7 (16.3%)	
	Middle	11 (25.6%)	25 (58.1%)	
	High	9 (20.9%)	11 (25.6%)	

HG: hyperemesis gravidarum.

**Figure 1:**
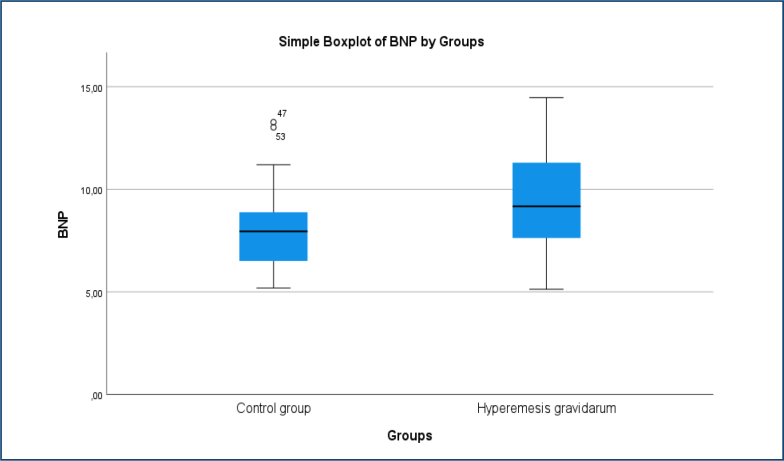
Comparison of BNP Levels Between Control Group and Hyperemesis Gravidarum Patients.

**Figure 2:**
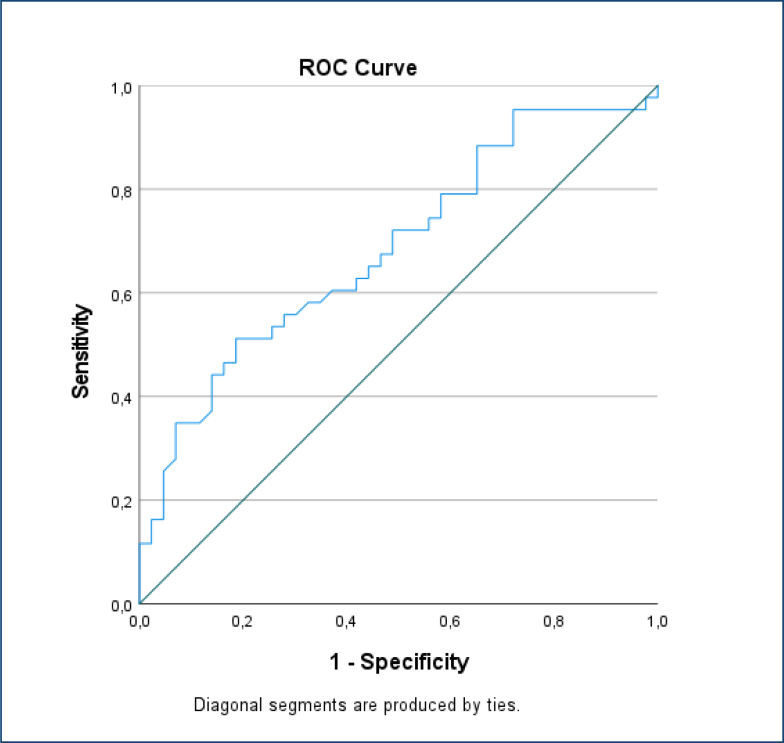
Receiver operating characteristic curve analysis of B-type natriuretic peptide levels.

The hemogram parameters, fasting blood glucose, urea, creatinine, AST, ALT, sodium, potassium, chloride, calcium, CRP, and TSH levels were statistically similar between the groups. Serum BNP levels were significantly higher in the HG cases group (HG group: 9.6±2.5 pg/mL [95%CI 8.9–10.3], control group 8.1±1.8 pg/mL [95%CI 7.5–8.6], Cohen's d=0.70, p=0.016) (Table 2, Figure 1). The diagnostic performance of BNP in distinguishing HG cases from healthy controls was assessed using receiver operating characteristic analysis. The area under the curve for BNP was 0.69 (95%CI 0.57–0.80, p=0.003), indicating good discriminative ability. The optimal cut-off value for BNP was 8.49 pg/mL, with a sensitivity of 60.5% and specificity of 60.5% (Figure 2). No significant correlation was observed between BNP and gestational week, age, BMI, or hemoglobin.

**Table 2:** Comparison of hemogram parameters and other biochemical values between control and experimental groups.

Variable	Control (n=43)	HG (n=43)	p-value
Wbc (10^3^/μL)	8.3±2.0	8.7±2.0	0.780
Neu (10^3^/μL)	5.5±1.7	6.1±1.9	0.558
Lym (10^3^/μL)	2.0±0.5	1.9±0.5	0.434
Hb (g/dL)	12.1±1.0	12.1±0.9	0.549
Hct (%)	36.1±2.5	35.8±0.9	0.339
Rdw-cv (%)	15.8±1.7	16.1±3.1	0.417
Mcv (fl)	80.9±6.2	79.3±9.8	0.335
Mch (pg)	26.0±3.0	27.2±2.4	0.006
Mchc (g/dL)	33.2±1.0	33.2±3.0	0.265
Plt (10^3^/μL)	240.0±1.7	227.7±39.7	0.103
Mpv (fl)	7.5±1.51	7.4±1.0	0.035
Pct (%)	0.2±0.0	0.2±0.0	0.036
TSH (μIU/mL)	1.6±1.0	1.6±1.5	0.174
FBG (mg/dL)	88.6±20.1	90.3±17.3	0.759
BUN (mg/dL)	7.9±2.0	7.9±2.5	0.367
Üre (mg/dL)	19.4±4.7	18.7±5.3	0.585
Cr (mg/dL)	0.6±0.0	0.6±0.1	0.324
AST (U/L)	13±0.0	13.1±1.0	0.013
ALT (U/L)	13.7±8.4	13.0±6.6	0.063
Na (mEq/L)	136.3±1.2	136.7±1.4	0.307
K (mEq/L)	3.8±0.2	3.8±0.2	0.585
Cl (mEq/L)	106.7±1.8	105.8±2.2	0.086
Ca (mg/dL)	9.4±0.6	9.5±0.6	0.575
CRP (mg/L)	5.9±7.8	5.9±6.4	0.713
BNP (pg/mL)	8.1±1.8	9.6±2.5	0.016

HG: hyperemesis gravidarum; Wbc: white blood cell; Neu: neutrophil; Lym: lymphocyte; Hb: hemoglobin; Hct: hematocrit; Rdw-cv: red cell distribution width − coefficient of variation; Mcv: mean corpuscular volume; Mch: mean corpuscular hemoglobin; Mchc: mean corpuscular hemoglobin concentration; Plt: platelet; Mpv: mean platelet volume; Pct: plateletcrit; TSH: thyroid stimulant hormone; BUN: blood urea nitrogen; Cr: creatinine; AST: aspartate aminotransferase; ALT: alanine aminotransferase; Na: sodium; K: potassium; Cl: chloride; Ca: calcium; CRP: C-reactive protein; BNP: B-type natriuretic peptide; FBG: fasting blood glucose.

## DISCUSSION

In our study, we found that serum BNP levels were significantly higher in the HG group compared to healthy pregnant women. Pregnancy is a physiological state of rapid volume expansion, whereas HG is characterized by rapid volume loss. Hypovolemia leads to a decrease in ventricular preload due to reduced intravascular volume, which in turn results in a decrease in ventricular filling pressure. In response to hypovolemia, ventricular strain increases to enhance cardiac output, placing an additional load on the heart and creating myocardial stress, which triggers BNP release^22–24^. Compensatory mechanisms in response to hypovolemia may lead to cardiac stress, which in turn could result in an increase in BNP levels.

In a normal pregnancy, BNP levels are approximately twice as high as in non-pregnant women, and they do not fluctuate significantly during pregnancy or in the postpartum period (4–6 weeks post-delivery)^25^. A recent study conducted on 405 women showed that BNP levels increased by approximately 1.8 times immediately after delivery. This increase in BNP levels was associated with chamber enlargement and the progression of hemoglobin reduction. These findings suggest that the increase in BNP levels may be due to left ventriclestretch caused by the increase in plasma volume and cardiac strain during delivery due to bleeding^26,27^. In our current study, we hypothesized that BNP levels increased due to cardiac strain caused by volume loss associated with HG following the physiological volume expansion during pregnancy. Additionally, in our study, in contrast to this previous study, no significant correlation was found between BNP levels and hemoglobin levels (p=0.254).

In patients with HG, morbidity can be associated with both maternal and fetal outcomes. This can lead to nutritional deficiencies, electrolyte imbalances, and complications such as Wernicke's encephalopathy. Additionally, esophageal trauma, cerebrovascular spasm, thrombosis, and psychological disorders may be observed^3^. Recent research has also highlighted the role of a disintegrin and metalloproteinase with thrombospondin motifs-1 (ADAMTS-1), a protease involved in extracellular matrix remodeling, in HG. ADAMTS-1 levels were found to be significantly elevated in HG patients compared to healthy pregnant women, suggesting a potential link between trophoblastic invasion and disease severity. Additionally, ADAMTS-1 showed a positive correlation with ketonuria, neutrophil count, and platelet distribution width (PDW), while being negatively correlated with mean corpuscular volume and TSH levels. These findings indicate that ADAMTS-1 might serve as an inflammatory and placental biomarker for HG, similar to the way BNP reflects cardiac stress^28^. Future studies should explore the combined role of BNP and ADAMTS-1 in predicting adverse outcomes in HG patients. Research on cardiovascular complications in HG patients is quite limited, and most studies are case reports. In these studies, cardiovascular complications are rare, but they are primarily due to electrolyte disturbances, particularly hypokalemia, hypomagnesemia, and hypocalcemia. These disturbances can lead to QTc prolongation and ventricular arrhythmias^29^. In our study, no significant electrolyte disturbances were observed, and no correlation was found between BNP and electrolyte levels. In patients with HG leading to electrolyte disturbances, BNP levels could be elevated due to cardiac stress, and BNP values might help predict these situations.

Inadequate adaptation to pregnancy-specific volume and hemodynamic changes may result in the development of hypertensive disorders of pregnancy^30^. In HG women, the risk of placental disorders and preeclampsia is increased. A 13-year cohort study showed a slight increase in the risk of preeclampsia in HG patients during the first trimester and a more than twofold increase in the risk of preeclampsia in women presenting with HG for the first time in the second trimester^31^. It has been reported that maternal serum BNP levels are higher in preeclamptic pregnancies compared to healthy pregnant women^32^. A very recent case-control study evaluating the relationship between cardiovascular biomarkers in pregnancy and preeclampsia suggested that BNP is a strong predictive biomarker for late-onset preeclampsia^33^. In our study, we found higher BNP levels in HG patients. Hemodynamic changes that hinder appropriate adaptation and lead to cardiac stress in HG patients may trigger the onset of preeclampsia. The early elevation of BNP levels should be investigated as a potential biomarker for predicting preeclampsia.

### Limitations

In our study, BNP levels after hydration were not assessed, so changes in BNP levels post-treatment could not be evaluated. Additionally, the lack of data on pregnancy complications (such as preeclampsia, gestational diabetes, etc.) in patients with elevated BNP levels is a limitation of our study. In future studies, it would be appropriate to evaluate BNP levels after treatment with a larger patient population and assess them in relation to the development of pregnancy complications such as preeclampsia.

## CONCLUSION

Our findings support the hypothesis that BNP may serve as a candidate biomarker for cardiac stress in HG patients, offering potential insights into the cardiovascular changes associated with this condition. However, further studies are required to confirm the role of BNP as a predictive tool for pregnancy-related complications and to evaluate its utility in monitoring the progression of HG. We recommend future longitudinal studies that track BNP levels over the course of pregnancy to assess its potential as a clinical marker for managing HG and preventing associated risks. Additionally, intervention-based studies could explore the impact of BNP monitoring on treatment outcomes. Acknowledging the limitations of our study, such as the lack of post-treatment BNP data and the absence of information on pregnancy complications like preeclampsia, future research should address these gaps to provide a more comprehensive understanding of BNP's clinical applicability.

## Data Availability

The datasets generated and/or analyzed during the current study are available from the corresponding author upon reasonable request.
